# Genome-wide identification and characterization of the ALBA gene family in rapeseed (*Brassica napus* L.) and its role in development and abiotic stress responses

**DOI:** 10.3389/fpls.2025.1721794

**Published:** 2026-01-05

**Authors:** Penghui Xu, Zhenzhong Chen

**Affiliations:** 1 School of Bioengineering, Xianning Vocational Technical College, Xianning, China; 2 College of Biology and Agricultural Resources, Huanggang Normal University, Huanggang, China

**Keywords:** abiotic stress, ALBA gene family, cis-acting regulatory elements, evolution, expression profiling

## Abstract

ALBA proteins (Acetylation lowers binding affinity) are an ancient group of DNA- and RNA-binding proteins found in archaea, bacteria, and eukaryotes. These small, basic, dimeric proteins perform diverse functions, including roles in RNA metabolism, transcriptional and translational regulation, and stress responses. In this study, 29 ALBA-coding genes were identified for the first time in the rapeseed genome, showing an uneven chromosomal distribution. Evolutionary analyses classified these genes into two subfamilies, RPP-20 and RPP-25, with the latter characterized by longer sequences due to the presence of RGG-box domains. Exon–intron structures and conserved motifs displayed distinct patterns between the two subfamilies. Segmental/whole genome duplication (WGD) were the primary forces driving the expansion of this gene family, while paralogous gene pairs were found to be under purifying selection, indicating conservation of their functional roles throughout evolution. Strong collinearity was also observed with Arabidopsis, *B. rapa*, and *B. oleracea*. The presence of *cis*-acting regulatory elements (CAREs) related to stress and hormone responses, growth and development, and circadian regulation, along with post-transcriptional regulation by miRNAs, suggests that *BnALBA* genes are subject to complex regulatory control and play essential roles in rapeseed growth and environmental adaptation. Transcriptome data from multiple tissues and developmental stages revealed distinct expression profiles, with some genes showing low expression, others being specifically activated in certain tissues or stages, and some displaying overlapping expression patterns. qRT-PCR analysis further confirmed that *BnALBA* genes respond significantly to abiotic stresses, with *BnALBA9* and *BnALBA22* being strongly induced under salt stress, while *BnALBA5*, *BnALBA9*, and *BnALBA27* showed substantial and consistent upregulation under drought stress. Together, these findings provide the first comprehensive identification and characterization of the *ALBA* gene family in rapeseed, underscoring their critical roles in regulating growth, development, and stress responses. This work establishes a foundation for future research aimed at improving rapeseed yield and resilience under adverse environmental conditions.

## Introduction

The diversity of abiotic stresses, such as drought, salinity, heat, and cold, can disrupt plant growth and development, leading to a significant decline in crop yield (Wai et al., 2021). Plants respond to abiotic stress factors through molecular mechanisms, which are considered the most complex processes, relying on the induction and regulation of transcription in stress-related genes (Shinozaki and Yamaguchi-Shinozaki, 2007). Through metabolic and gene expression reprogramming, plants develop tolerance to various abiotic stress factors, thereby establishing a balance among all plant components essential for optimal function (Muñoz et al., 2016). In this context, evolutionarily conserved DNA-binding proteins, such as histones and transcription factors, function as on-off switches for genes (Choudhary et al., 2009). Another group of DNA-binding proteins includes the Alba (Acetylation lowers binding affinity) family proteins.

Alba proteins, which belong to the sequence-independent DNA-binding proteins, constitute an ancient group that emerged before the divergence of archaea and eukaryotes. These primarily basic proteins form dimers and possess a remarkably high affinity for double-stranded DNA, which underlies their designation as Acetylation lowers binding affinity (Alba) (Bell et al., 2002; Črnigoj et al., 2011; Goyal et al., 2016). Beyond DNA, they also interact with various RNA molecules (Xue et al., 2000). Alba proteins modulate their activity via acetylation by PAT (protein acetyltransferase) and deacetylation by Sir2 (an NAD+-dependent histone deacetylase, HDAC), a regulatory mechanism similar to histone-mediated transcriptional control (Bell et al., 2002; Zhao et al., 2003). Furthermore, the ubiquitous presence of Alba family proteins across diverse organisms highlights their involvement in multiple cellular processes, including transcriptional and translational regulation, chromatin dynamics, and growth and development (Goyal et al., 2016). Indeed, most organisms express at least two proteins containing the Alba domain (Jagadeesh and Vembar, 2024).

The Alba protein family is categorized into three primary groups: archaeal Alba proteins, represented by the SsH10b protein from *Sulfolobus shibatae* (Aravind et al., 2003); eukaryotic Alba proteins present in both unicellular and multicellular organisms, encompassing the P20 family (Rpp20, a subunit of RNase P, also called Pop7 in budding yeast) and the P25 family (Rpp25, another RNase P subunit designated as Pop6) (Chan et al., 2018; Yin et al., 2021).

These proteins contain an Alba domain comprising more than 90 amino acids (Goyal et al., 2016). This highly conserved domain facilitates nucleic acid binding. Alba proteins additionally feature an IF3-C-like structure that enables RNA binding (Aravind et al., 2003). The Alba domain manifests in several configurations, including small proteins with a single generic Alba domain found in all archaeal Alba homologs, larger proteins containing one Alba domain as seen in eukaryotic Alba proteins of the Rpp20/Pop7 group, and more complex proteins that incorporate additional RGG/RG repeat motifs or extra domains, which characterize eukaryotic Alba proteins of the Rpp25/Pop6 group (Aravind et al., 2003; Goyal et al., 2016; Náprstková et al., 2021). The RGG/RG repeat motifs are implicated in diverse cellular processes including DNA damage response, snRNP biogenesis, apoptosis regulation, transcription, mRNA splicing, and translation - with many of these functions being modulated, at least partially, through arginine methylation of the RGG/RG motifs (Thandapani et al., 2013). The RGG box has been detected in Alba proteins across multiple species, encompassing *Trypanosoma brucei*, *Leishmania infantum*, *Toxoplasma gondii*, *Plasmodium falciparum*, *Plasmodium berghei*, Arabidopsis, and tomato (Verma et al., 2018; Náprstková et al., 2021; Wai et al., 2021).

Besides the Alba domain, these proteins can contain additional functional domains, such as the CLIP1 zinc finger in the nematode *Pristionchus*, the Dynactin p22 subunit, FAD-binding domains, F-box domains in the fungus *Taphrina*, NT5C domains in the oomycete *Pythium*, and the ATP synthase subunit H domain in the protozoan parasite *Theileria* (Aravind et al., 2003). The functional diversification and specialization of Alba proteins presumably stem from novel domain combinations acquired through recombination, duplication, and divergence (Verma et al., 2018). This evolutionary adaptability underlies the species-specific diversity and functional plasticity of the Alba protein family, allowing these proteins to accommodate the intricate and diverse requirements of cellular processes (Wai et al., 2021).

Environmental stresses trigger modifications in Alba protein behavior across diverse organisms (Dupé et al., 2015). *Plasmodium falciparum* encodes six Alba proteins, with four (*PfAlba1-4*) characterized to date. Their dual DNA/RNA-binding capability implies bifunctional roles in maintaining *P. falciparum* chromatin architecture and modulating RNA metabolism (Chêne et al., 2012). In *Trypanosoma brucei*, four identified Alba proteins demonstrate RNA-binding activity and accumulate in cytoplasmic stress granules (SGs) during nutritional stress (Mani et al., 2011). The *Leishmania infantum* proteins *LiAlba1* and *LiAlba3* associate with RNA-binding proteins and ribosomal subunits to mediate translational repression. Notably, these proteins undergo cytoplasmic-to-nucleolar translocation during heat stress response (Dupé et al., 2015; Matiz-González et al., 2024). *Toxoplasma gondii* expresses two Alba homologs (TgAlba1 and TgAlba2) showing dual nuclear-cytoplasmic distribution, suggesting essential functions in both cellular compartments (Olguin-Lamas et al., 2011). Gene knockdown experiments have established their participation in stress response modulation and developmental differentiation, where they interact with multiple RNA-binding proteins to regulate translational gene expression (Gissot et al., 2013).

In plants, multiple Alba domain-containing proteins have been identified, although their functional characterization remains limited (Jagadeesh and Vembar, 2024). Rice harbors nine *ALBA* genes displaying substantial expression variation in response to diverse abiotic stresses and phytohormonal treatments (Verma et al., 2018). The upregulation of *OsALBA1* under water deficit and oxidative stress implies potential involvement of Alba proteins in conferring rice tolerance to environmental challenges (Verma et al., 2014). Tomato genomes contain eight Alba-encoding genes exhibiting significant tissue-specific expression patterns and responsiveness to salinity, drought, heat stress, and ABA treatment (Wai et al., 2021). Cotton studies identified *GhALBA4* and *GhALBA5* as strongly induced under drought and salt stress conditions. Virus-induced gene silencing (VIGS) of these genes generated mutants displaying increased sensitivity to dehydration and salinity, supporting their functional relevance in abiotic stress tolerance (Magwanga et al., 2019). Arabidopsis possesses six Alba homologs (Wang et al., 2019). Investigations revealed that the ALBA domains in *AtAlba1* and *AtALBA2* mediate R-loop interactions, contributing to gene expression regulation, chromatin maintenance, and DNA repair mechanisms. In contrast, *AtAlba4* and *AtAlba6* function in RNA metabolism, male gametophyte development, and heat stress responses (Yuan et al., 2019; Náprstková et al., 2021). Significantly, moderate heat stress (37 °C) modulated expression profiles of most Arabidopsis *ALBA* genes in inflorescence tissues (Náprstková et al., 2021). Functional analysis of *AtALBA3* established its essential role in maintaining male fertility during heat stress through protection of pollen-specific mRNAs (Ci et al., 2025).

Rapeseed (*Brassica napus*), a member of the Cruciferae/Brassicaceae family, possesses a complex genome structure. This allopolyploid species evolved through natural hybridization between two diploid ancestors: *B. rapa* (AA genome, 2n=20) and *B. oleracea* (CC genome, 2n=18) (Song et al., 2020). As the third most significant global source of vegetable oil after soybean and palm oil, rapeseed serves diverse applications including biofuel production, human nutrition, animal feed, and utilization in chemical and pharmaceutical industries (Friedt et al., 2018). Nutritionally valuable rapeseed oil contains high levels of unsaturated fatty acids and is cholesterol-free. Its essential fatty acids, which humans cannot synthesize endogenously, play crucial roles in health maintenance (Hoffman and Gerber, 2014). Nevertheless, rapeseed cultivation consistently encounters challenges from multiple biotic and abiotic stresses induced by environmental variability, significantly impacting crop yield (Di et al., 2018). In this study, the *Alba* gene family was identified in rapeseed for the first time, and its evolutionary trajectory was thoroughly investigated. Furthermore, the expression patterns of these genes were analyzed across various rapeseed tissues during plant development using transcriptome data, and their responses to salt and drought stresses were assessed through qRT-PCR.

## Materials and methods

### Plant materials and experimental treatments

Seeds of rapeseed (cultivar ZS11) randomly selected for germination testing. Only seeds with a 100% germination rate were considered high-vigor and used for the experiment. These seeds were surface-sterilized with 10% sodium hypochlorite for 5 minutes. The sterilized seeds were sown on moist filter paper and germinated in a growth chamber set at 25 °C with a 16-hour light/8-hour dark photoperiod. After seven days, the uniform seedlings were transferred to containers with Hoagland’s nutrient solution, supported by foam plugs. The nutrient solution was renewed every other day until the plants reached the four-leaf stage. At this stage, stress treatments were initiated by transferring the plants to a modified Hoagland’s solution containing either 150 mM NaCl to induce salt stress or 20% (w/v) polyethylene glycol (PEG-6000) to simulate drought stress. While PEG-6000 and NaCl are widely accepted tools for inducing drought and salt stress under controlled conditions, it is recognized that they may not fully reproduce all aspects of field environments. PEG-6000 primarily imposes osmotic stress rather than actual soil drying (Kylyshbayeva et al., 2024), and NaCl treatments may differ from the complex ionic composition and gradual salt accumulation found in natural saline soils (Plessis, 2023). Nevertheless, both methods provide reliable and reproducible systems for investigating plant physiological responses to water deficit and salinity stress. True leaves were harvested at 0, 3, 6, 12, and 24 hours following stress treatment, immediately frozen in liquid nitrogen, and stored at -80 °C until further analysis (Wang et al., 2022; Xue et al., 2025).

### Identification of *ALBA* gene family members in rapeseed

To comprehensively identify the *ALBA* gene family in rapeseed, the reference genome of the cultivar Darmor-bzh (version Brana_Dar_v5), obtained from the BRAD V3.0 database, was used. The conserved ALBA domain Hidden Markov Model (HMM) profile (PF01918) was then retrieved from the Pfam 37.4 (Paysan-Lafosse et al., 2025). This profile was used to search the entire rapeseed proteome for proteins containing the ALBA domain using HMMER software version 3.4, with a threshold of E-value < 0.01 (Mistry et al., 2013). Candidate sequences were further verified by checking for the presence of the ALBA domain using the SMART v10 and NCBI Conserved Domain Database (Letunic et al., 2021; Wang et al., 2023). Finally, incomplete, redundant, or sequences lacking the complete domain were removed to produce a final set of validated *ALBA* family genes.

### Physicochemical properties of the *ALBA* gene family

To examine the structural and functional characteristics of ALBA proteins, essential physicochemical parameters such as molecular weight, isoelectric point (pI), instability index, aliphatic index, and grand average of hydropathicity (GRAVY) were calculated using the Multiple Protein Profiler 1.0 (MPP) server (Sganzerla Martinez et al., 2024). Additionally, subcellular localization predictions were performed using the DeepLoc 2.1 server (Ødum et al., 2024).

### Protein conservative domain and gene architecture analysis of *BnALBAs*

The rapeseed genome GFF3 file was first downloaded from BRAD V3.0 and then used to analyze the genomic organization of the *ALBA* genes, including the number, length, and distribution of exons and introns Gene structures were visualized and analyzed using TBtools-II software (Chen et al., 2023). Additionally, conserved protein motifs were identified using the MEME suite v 5.5.8 with the maximum number of motifs set to 15, motif lengths constrained to between 6 and 100 amino acids, and E. value < 0.05 (Bailey et al., 2015).

### Multiple sequence alignment and construction of phylogenetic tree of *BnALBA* gene family

To investigate the evolutionary relationships of *ALBA* genes in rapeseed and their phylogenetic links with other plant species, protein sequences of rapeseed *ALBAs* and their homologs from six species including Arabidopsis (*AtALBA*), rice (*OsALBA*), sorghum (*SbALBA*), potato (*StALBA*), wheat (*TaALBA*), and soybean (*GmALBA*) were obtained for analysis. Multiple sequence alignment was performed using ClustalW (Larkin et al., 2007). A phylogenetic tree was then constructed using the Neighbor-Joining (NJ) method in MEGA12 software with 1000 bootstrap replicates to assess node support (Kumar et al., 2024). The resulting tree was visualized using the interactive Tree of Life (iTOL) platform, v6.0 (Letunic and Bork, 2024).

### Analysis of promoter *cis*-acting regulatory elements and miRNA target prediction

To identify *cis*-regulatory acting elements (CAREs) involved in transcriptional control, a 1.5 kb genomic sequence upstream of the translation start site (ATG) of each *ALBA* gene was retrieved using the BioMart tool in the Ensembl Plants database and analyzed using the PlantCARE web server (Lescot et al., 2002; Kinsella et al., 2011). The analysis focused on detecting five major categories of CAREs, namely circadian-related elements, tissue- and development-specific elements, hormone-responsive elements, core promoter and transcription factor binding sites, and stress-responsive elements. For the prediction of post-transcriptional regulation, potential microRNA (miRNA) target sites within the *ALBA* gene sequences were identified using the psRNATarget v2 platform, with the following parameters: maximum expectation value = 3.5, complementarity scoring length (hsp size) = 21 bp, target accessibility (UPE) = 25, flanking sequence lengths of 17 bp upstream and 13 bp downstream, and a translational inhibition range of 9–11 nucleotides (Dai et al., 2018).

### Gene duplication, synteny, and Ka/Ks analysis of *BnALBAs*

To identify the duplication modes of *BnALBA* genes, intra-genomic and inter-genomic synteny analyses were conducted between rapeseed and related species (*Arabidopsis thaliana*, *Brassica rapa*, and *Brassica oleracea*) using MCScanX with the following parameter settings: MATCH_SCORE = 50; MATCH_SIZE = 5; GAP_PENALTY = −1; OVERLAP_WINDOW = 5; E_VALUE = 1 × 10^-5^; and MAX_GAPS = 25. The resulting syntenic relationships and duplication events were visualized using TBtools. To assess evolutionary selection pressures, non-synonymous (Ka) and synonymous (Ks) substitution rates were calculated for duplicated *ALBA* gene pairs using the KaKs_Calculator implemented in TBtools. The Ka/Ks ratio for each pair was interpreted as follows: Ka/Ks < 1 indicates purifying selection, Ka/Ks = 1 suggests neutral evolution, and Ka/Ks > 1 implies positive selection.

### Expression pattern analysis of rapeseed *ALBA* genes using transcriptomic data

To investigate the expression profiles of *ALBA* gene family members across various developmental stages in rapeseed, transcriptomic data were obtained from the Electronic Fluorescent Pictograph (EFP) browser of the BrassicaEDB database (Chao et al., 2020). The dataset encompassed expression levels of *ALBA* genes in multiple tissues during key developmental phases: seedling, bolting, initial flowering, full-bloom, podding, and maturation. A heatmap visualization was generated based on log2 (FPKM + 1) transformed values using TBtools-II software (Chen et al., 2023).

### RNA extraction, reverse transcription, and qRT-PCR analysis

Total RNA was extracted using the FastPure Universal Plant Total RNA Isolation Kit (Vazyme, Nanjing, China) following the manufacturer’s protocol. RNA concentration was measured using a NanoDrop 2000C spectrophotometer (Thermo Fisher Scientific, USA), while integrity was verified by 1% agarose gel electrophoresis. First-strand cDNA was synthesized from 1 μg of total RNA using the EasyScript One-Step gDNA Removal and cDNA Synthesis SuperMix (TransGen, Beijing, China). Quantitative real-time PCR (qRT-PCR) was performed using TB Green Premix Ex Taq II (Tli RNaseH Plus; TaKaRa) on an ABI 7500 Real-Time PCR System (Applied Biosystems, USA). The thermal cycling conditions comprised initial denaturation at 95 °C for 5 min, followed by 40 cycles of 95 °C for 10 s and 60 °C for 30 s. The *BnActin7* gene served as an internal control for normalization, and relative expression levels were calculated using the 2^−ΔΔCt^ method (Livak and Schmittgen, 2001). Gene-specific primers were designed using Primer-BLAST (**Supplementary File 1**).

### Statistical analysis

Experiments were conducted with three biological replicates, each comprising three technical replicates. Gene expression differences between treatments and controls were assessed using Student’s t-test, with statistical significance defined at p < 0.05, p < 0.01, and p < 0.001. Error bars represent standard deviation.

## Results

### Identification of the *ALBA* gene family in rapeseed

This study employed bioinformatics analyses to investigate the *ALBA* gene family in rapeseed, identifying 29 genes containing the ALBA domain within its genome. Except for the BnALBA15, which is localized to the endoplasmic reticulum, the proteins encoded by the other ALBA proteins are predicted to localize to the cytoplasm/nucleus (**Supplementary File 2**). Physicochemical characterization revealed substantial diversity among the BnALBA proteins in terms of length, molecular weight, isoelectric point (pI), grand average of hydropathicity (GRAVY), aliphatic index, and instability index (**Supplementary File 2**). The average protein length was 239.9 amino acids, with an average molecular weight of 26.07 kDa and an average pI of 7.87. Mean values for GRAVY, aliphatic index, and instability index were -0.56, 73.6, and 40.78, respectively. Among these proteins, the shortest sequences were observed in BnALBA12 and BnALBA25 (123 aa), while BnALBA15 was the longest (583 aa). GRAVY values ranged from -1.2 in BnALBA22 and BnALBA27 to 0.04 in BnALBA25. The aliphatic index varied from 41.46 in BnALBA22 to 102.28 in BnALBA25, and the instability index ranged from 30.08 in BnALBA25 to 51.82 in BnALBA2. Molecular weights spanned from 13.46 kDa in BnALBA25 to 64.18 kDa in BnALBA15, while pI values exhibited a range from 5.58 in BnALBA13 to 9.87 in BnALBA12 and BnALBA15 (**Supplementary File 2**).

### Phylogenetic analysis of BnALBA proteins in rapeseed

**Figure 1:**
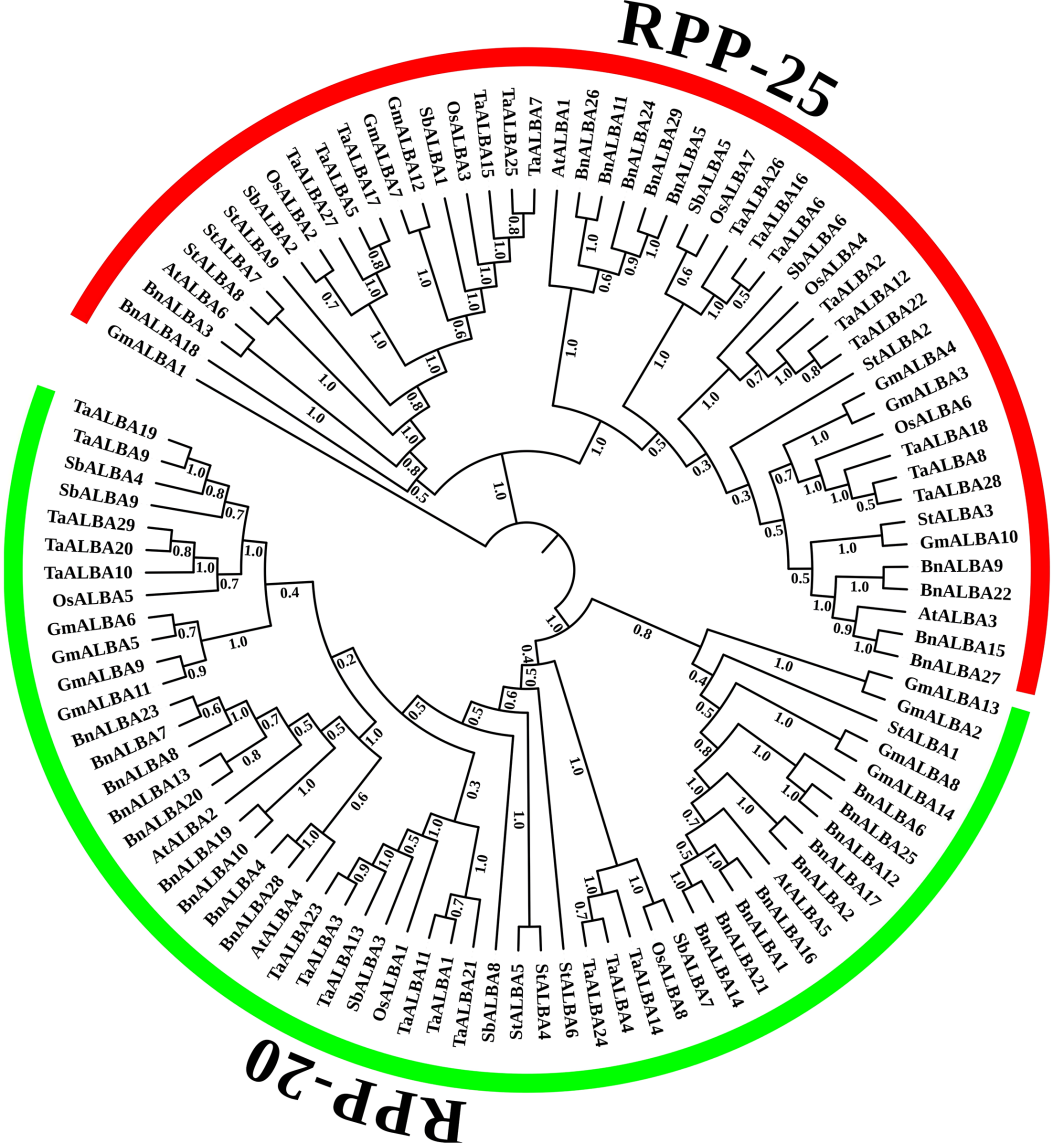
Phylogenetic tree of the *ALBA* gene family in rapeseed. A total of 104 full-length ALBA proteins from rapeseed (*BnALBA*), rice (*OsALBA*), Arabidopsis (*AtALBA*), sorghum (*SbALBA*), potato (*StALBA*), and soybean (*GmALBA*) were aligned using ClustalW. The evolutionary tree was constructed using the Neighbor-Joining (NJ) algorithm in MEGA12 software and visualized with iTOL v6.0. Subfamilies RPP-20 and RPP-25 are highlighted in green and red, respectively.

To elucidate the evolutionary relationships among *ALBA* genes across species, a phylogenetic tree was constructed using the Neighbor-Joining method based on protein sequences from 14 *GmALBA* (soybean), 29 *BnALBA* (rapeseed), 6 *AtALBA* (Arabidopsis), 9 *StALBA* (potato), 9 *SbALBA* (sorghum), 8 *OsALBA* (rice), and 29 *TaALBA* (wheat) genes (**Figure 1**). The analysis revealed a clear division of *ALBA* genes into two distinct subfamilies. The RPP-25-like subfamily contained 49 members, including 6 *GmALBA*, 11 *BnALBA*, 3 *AtALBA*, 5 *StALBA*, 4 *SbALBA*, 5 *OsALBA*, and 15 *TaALBA* genes. The RPP-20-like subfamily comprised 55 genes, including 8 *GmALBA*, 18 *BnALBA*, 3 *AtALBA*, 4 *StALBA*, 5 *SbALBA*, 3 *OsALBA*, and 14 *TaALBA* genes. This phylogenetic division suggests significant evolutionary divergence and potential functional differentiation between the two subfamilies (**Figure 1**).

### Conserved motif and gene structure analysis of *BnALBAs*

**Figure 2:**
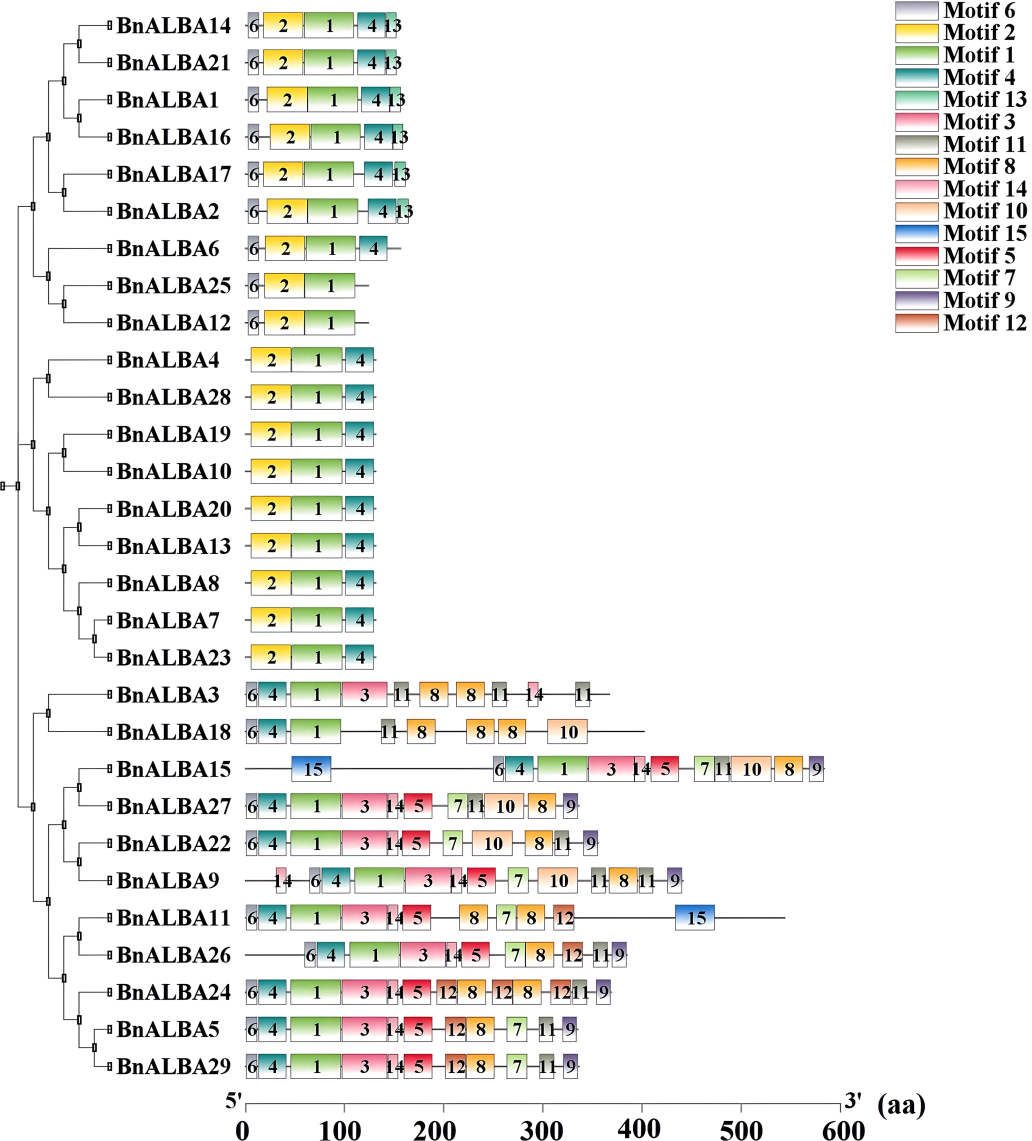
Conserved motifs in rapeseed ALBA proteins. Fifteen conserved motifs of varying lengths and frequencies were identified. Each motif is represented as a colored box with a unique number. A scale bar is shown at the bottom of the figure, indicating the relative lengths in amino acids (aa). Color codes correspond to individual motifs. Visualization was performed using TBtools software.

**Figure 3:**
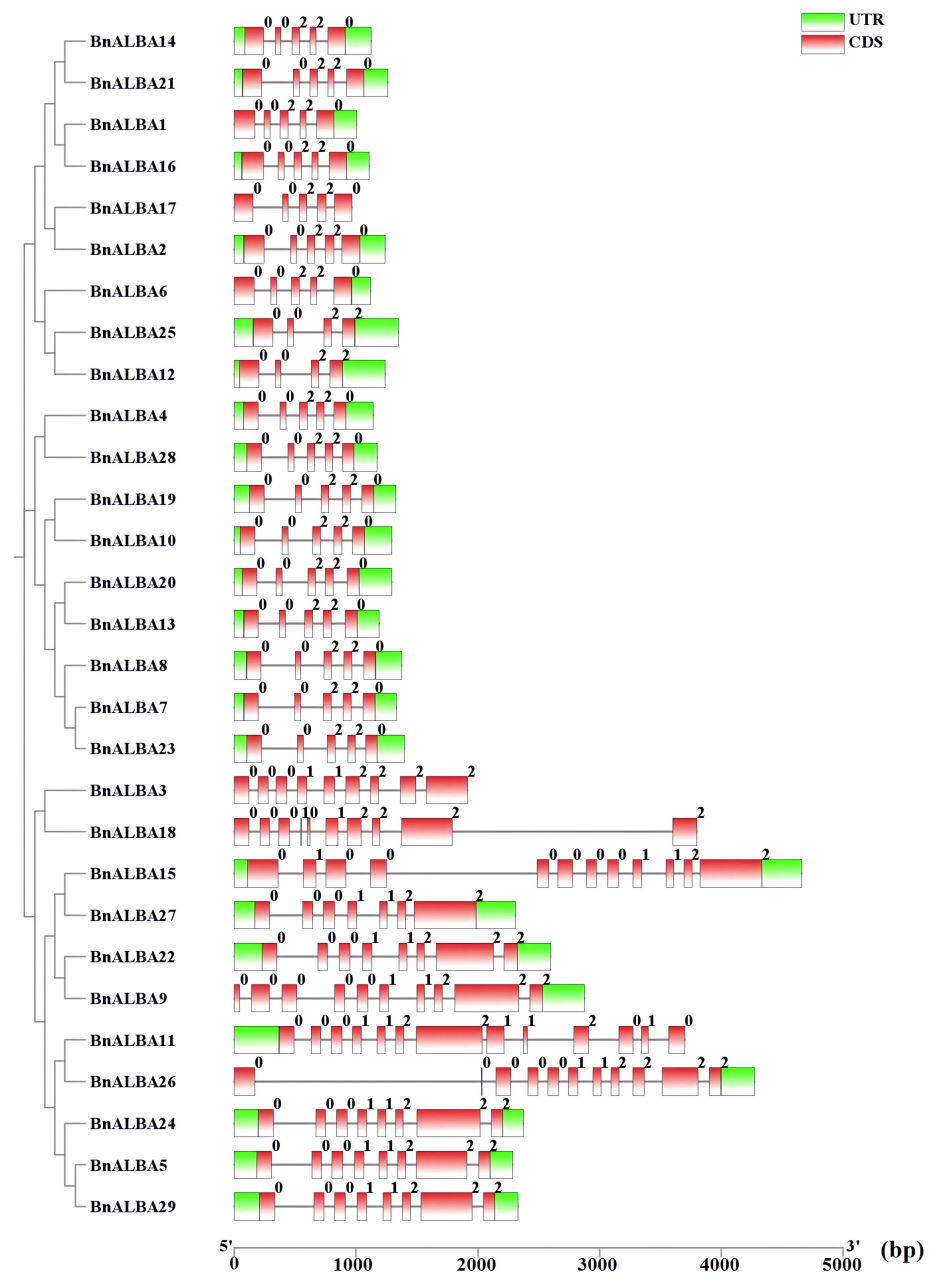
Exon–intron structure of the *BnALBA* gene family. Gene structures are illustrated with green boxes for UTRs, red boxes for exons, and black lines for introns. Intron phases are indicated by numbers (0, 1, and 2). A scale bar is shown at the bottom of the figure, indicating relative lengths in nucleotides (bp). Visualization was performed using TBtools software.

Protein motif conservation within the *BnALBA* gene family was analyzed using the MEME tool (**Supplementary File 3**). Fifteen conserved motifs were identified, among which Motif 1, containing the characteristic ALBA domain, was universally conserved across all 29 BnALBA proteins, indicating its fundamental functional role (**Supplementary File 3**; **Figure 2**). The remaining motifs exhibited variable distribution patterns across different members. Notably, RGG/RG repeat-containing motifs were exclusively identified in members of the RPP25 subfamily, emphasizing their specific association with RNA-binding activity (**Supplementary File 3**; **Figure 2**).Exon-intron structure analysis revealed distinct organizational patterns between the two phylogenetic groups. The RPP-20 group contained genes with 3–4 introns, while the RPP-25 group exhibited substantially more complex gene structures with 6–12 introns (**Figure 3**). Correspondingly, RPP-25 genes were generally longer than RPP-20 genes. Within the RPP-25 group, several genes showed distinctive features: *BnALBA15* possessed an elongated fourth intron, *BnALBA26* contained an extended first intron, *BnALBA18* featured a lengthened ninth intron, and *BnALBA11* displayed the most complex structure with twelve introns (**Figure 3**).

### Chromosome mapping, synteny analysis and selection pressure

**Figure 4:**
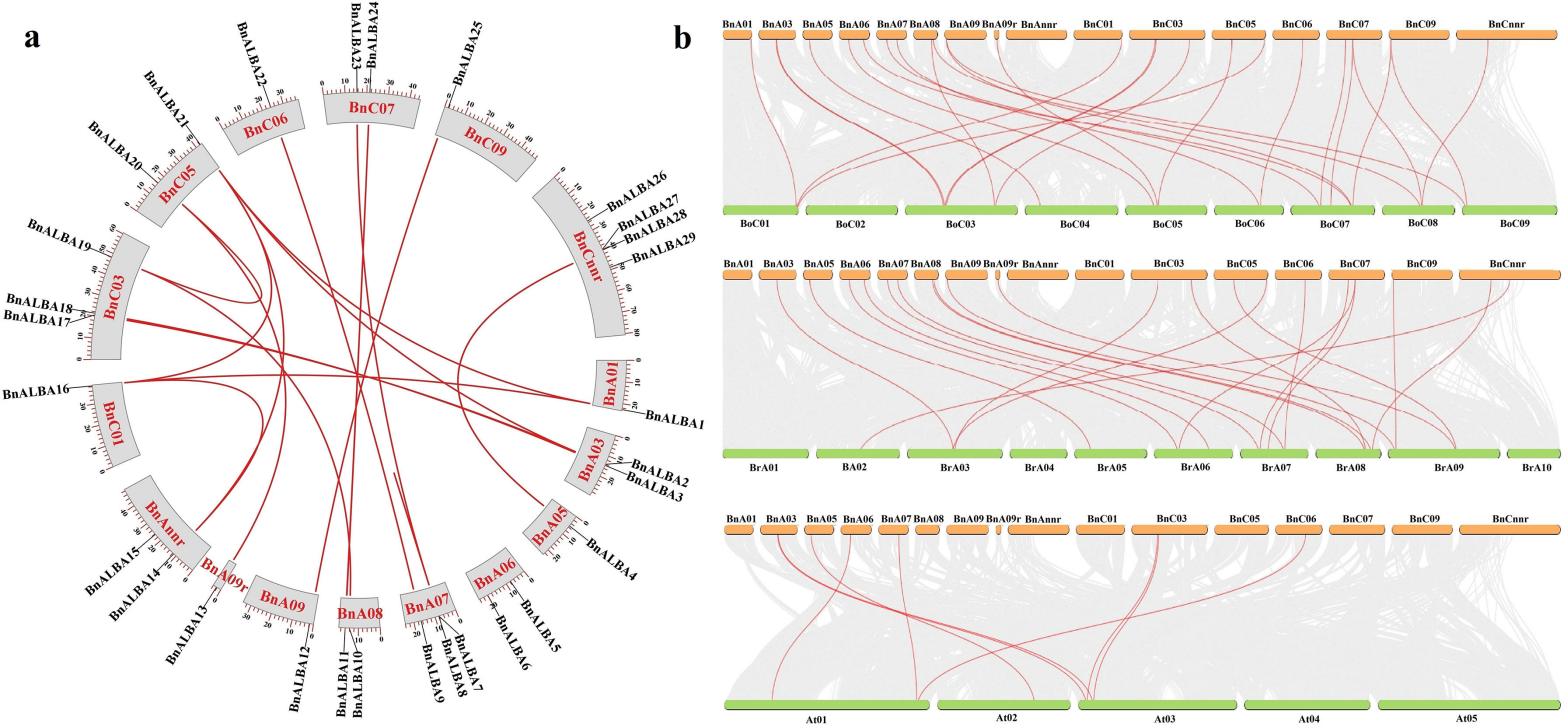
Chromosomal locations and collinearity analysis of *BnALBA* genes within and between species. **(a)** The circular diagram illustrates the chromosomal positions of *BnALBA* genes in rapeseed. Red curves represent 17 paralogous gene pairs. **(b)** Syntenic relationships between rapeseed and Arabidopsis, *B*. *rapa*, and *B*. *oleracea*. Gray lines indicate overall collinear relationships, while red lines mark collinear *ALBA* genes. The diagrams were generated using TBtools software.

The genomic distribution of *BnALBA* genes revealed an uneven pattern across subgenomes, with twelve genes located on the An subgenome chromosomes, ten on the Cn subgenome chromosomes, and seven on scaffold regions of the genome assembly (**Figure 4a**). Chromosomal distribution analysis showed the highest gene density on chromosomes A07 and C03 (three genes each), while chromosomes A01, A05, A09, C01, C06, and C09 each contained a single gene. An intermediate distribution of two genes per chromosome was observed on chromosomes A03, A06, A08, C05, and C07 (**Figure 4a**).

Investigation of the expansion mechanisms of the *ALBA* gene family in rapeseed identified multiple duplication events, with *BnALBA15* and *BnALBA29* originating through dispersed duplication, *BnALBA8* arising from tandem duplication, and the remaining genes resulting from segmental duplication events. These findings indicate that segmental duplication served as the primary mechanism for the expansion and diversification of the *BnALBA* gene family.

Collinearity analysis identified 17 paralogous gene pairs within the rapeseed genome (**Figure 4a**). Interspecies synteny analysis revealed extensive evolutionary relationships, with 8 orthologous pairs identified between rapeseed and Arabidopsis, 26 pairs with *B. oleracea*, and 21 pairs with *B. rapa*, demonstrating substantial genomic conservation with closely related species (**Figure 4b**). To assess evolutionary selection pressures, Ka and Ks substitution rates were calculated for paralogous gene pairs. The resulting Ka/Ks ratios showed an average value of 0.189, significantly less than 1, indicating that the *BnALBA* genes have predominantly undergone purifying selection throughout their evolution (**Supplementary File 4**). This pattern suggests strong functional constraints have maintained protein integrity while efficiently eliminating deleterious mutations.

### Predicted miRNA target site of the *BnALBA* gene family

Computational analysis of miRNA targeting patterns identified five miRNA molecules from three distinct families that potentially regulate the post-transcriptional expression of four *BnALBA* genes. Specifically, bna-miR162a was predicted to target *BnALBA3*, while the bna-miR172a/d group showed potential regulation of *BnALBA18* transcripts. Additionally, bna-miR6029 was identified as a putative regulator of both *BnALBA14* and *BnALBA21*. These findings suggest that these miRNAs may play significant roles in the post-transcriptional regulatory networks of the *BnALBA* gene family.

### CAREs analysis of promoter regions in *BnALBA* genes

**Figure 5:**
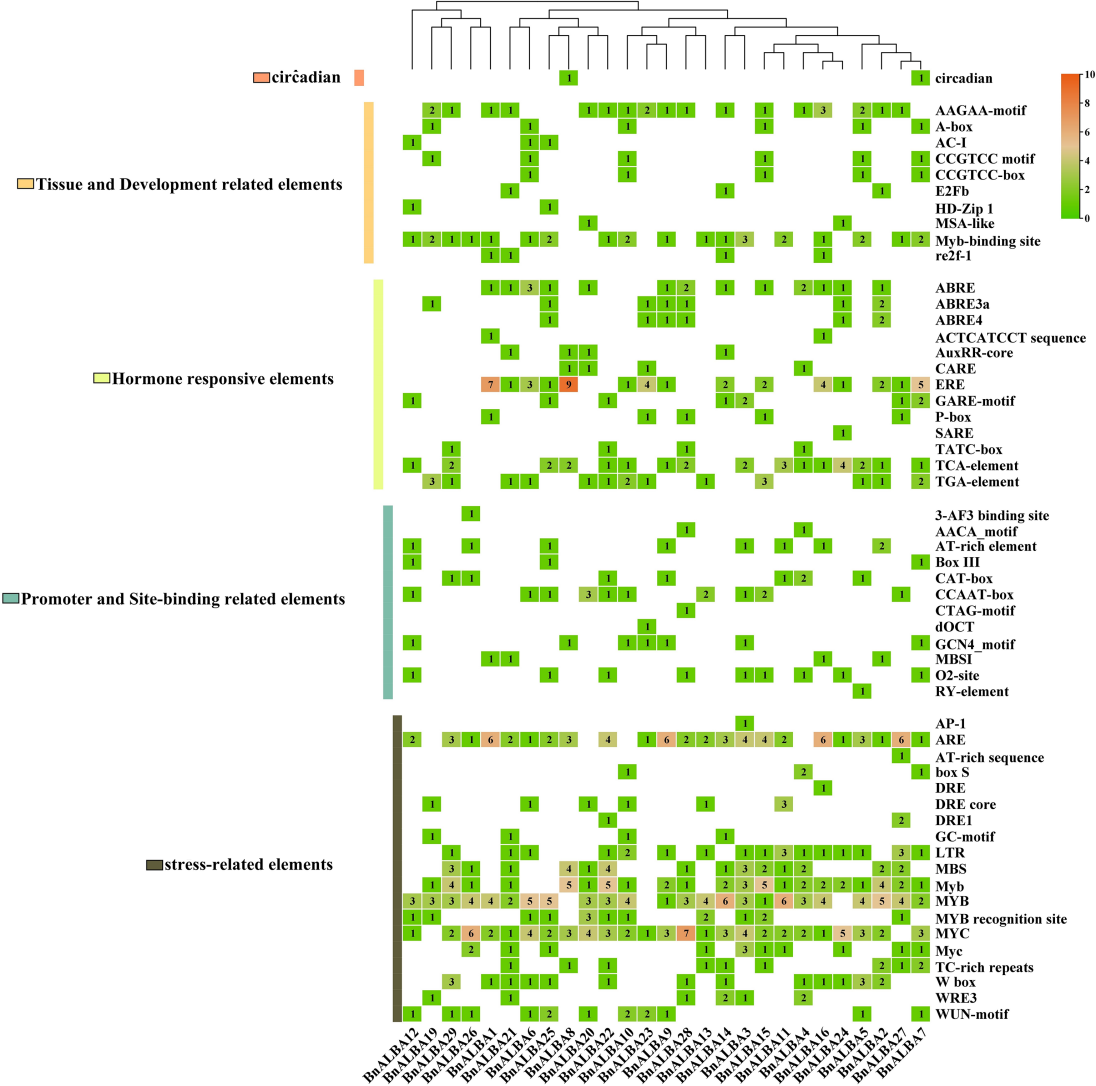
Promoter analysis of *BnALBA* genes. The heatmap displays the type and abundance of cis-regulatory elements identified in *BnALBA* genes.

*In silico* investigation of promoter regions in the *BnALBA* genes identified a comprehensive set of CAREs, with 709 elements categorized into five functional classes (**Figure 5**). Circadian-related elements were represented by a single type with a frequency of 2 (**Figure 5**). Tissue and development-related elements constituted 79 elements, encompassing 10 types involved in processes such as xylem formation, mesophyll cell differentiation, alpha-amylase regulation, and cell cycle control (**Figure 5**). Hormone-responsive elements formed the second largest group with 151 elements, including 13 types responsive to auxin, gibberellin, ethylene, abscisic acid, and salicylic acid (**Figure 5**). The promoter and site-binding related elements category contained 60 elements consisting of 12 types associated with CMA3 activity, ATBP-1 binding, MYBHv1 function, endosperm and meristem expression, flavonoid metabolism, zein synthesis, and seed development (**Figure 5**). Stress-responsive elements were the most abundant with 417 elements, comprising 19 types conferring resistance to cadmium, anaerobic conditions, mechanical injury, oxygen deficiency, low temperature, and drought (**Figure 5**).

Among the developmental elements, the AAGAA-motif and MYB-binding site showed the highest frequencies with 26 and 22 occurrences, respectively (**Figure 5**). The ERE element was most prevalent in hormone-responsive elements with 44 repeats. The CCAAT-box appeared most frequently in the promoter elements group with 14 occurrences, while MYB, MYC, and ARE elements dominated the stress-responsive group with 85, 69, and 66 repeats, respectively (**Figure 5**). *BnALBA3* was found to harbor the largest number of regulatory elements, totaling 35, whereas *BnALBA12* contained the smallest number with only 18 elements *BnALBA13* and *BnALBA23* contained 17 elements each, and other family members contained between 20 and 33 elements. These quantitative differences in CAREs composition suggest variations in regulatory complexity and potential functional specialization among *BnALBA* genes in responding to developmental cues, hormonal signals, and environmental stresses. The findings suggest that *BnALBA* genes may have a sophisticated regulatory system potentially capable of integrating diverse internal and external signals through their complex promoter architectures (**Figure 5**).

### Expression analysis of *BnALBA* genes based on RNA-seq data

**Figure 6:**
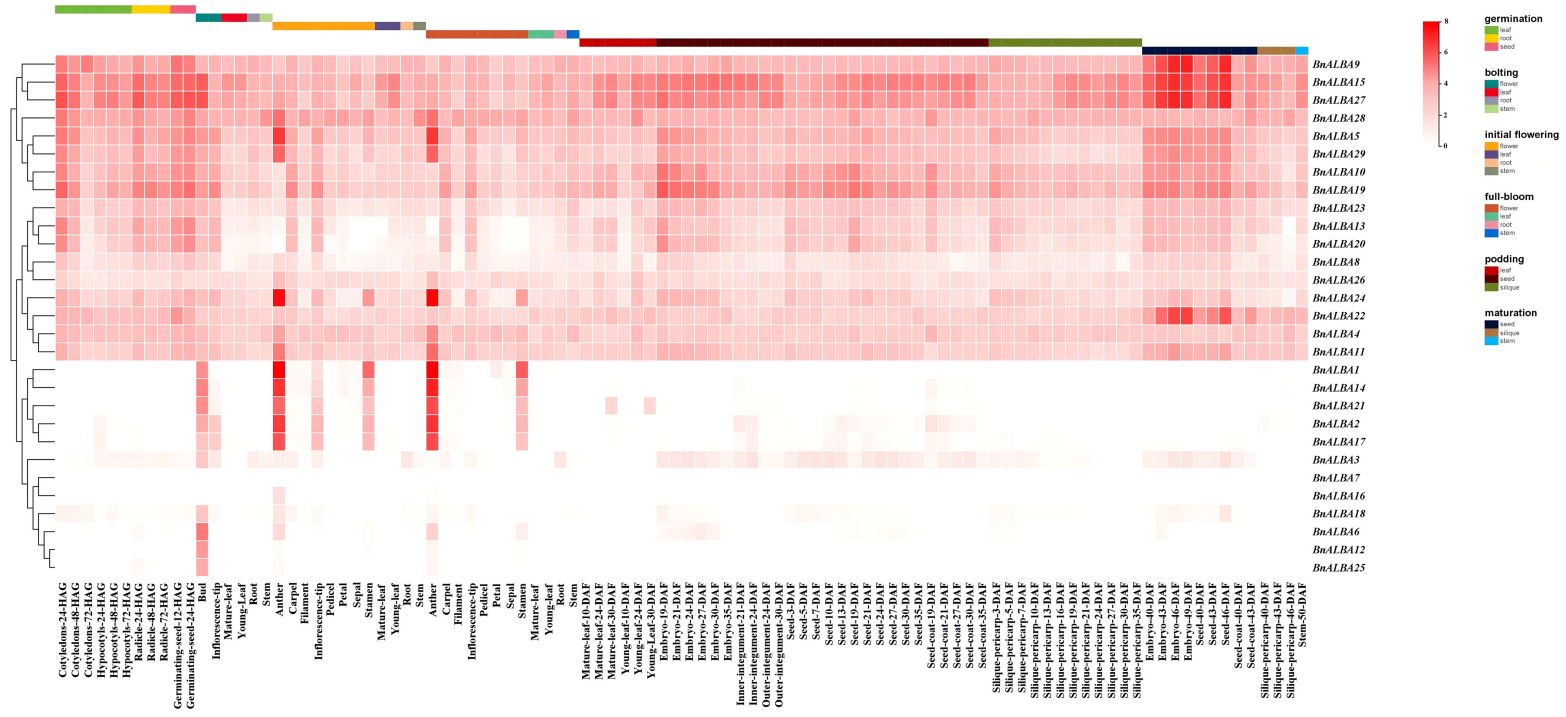
Expression profiles of *BnALBA* genes in rapeseed tissues across developmental stages, including seedling, bolting, initial flowering, full-bloom, podding, and maturation, based on RNA-seq data. Expression values are presented as Log2 (FPKM + 1). The heatmap was generated using TBtools software.

This study investigated the biological functions of the *BnALBA* gene family in rapeseed by analyzing the expression patterns of all 29 genes across six tissues, namely root, stem, leaf, flower, seed, and silique, and six developmental stages including germination, bolting, initial flowering, full-bloom, podding, and maturation, based on RNA-seq data (**Figure 6**). Analysis of expression patterns through heatmap visualization revealed substantial variation among genes, allowing their classification into three main categories. The first category included eight genes, *BnALBA5*, *BnALBA9*, *BnALBA10*, *BnALBA15*, *BnALBA19*, *BnALBA27*, *BnALBA28*, and *BnALBA29*, which displayed consistently high expression across most examined tissues (**Figure 6**). The second category comprised nine genes, *BnALBA4*, *BnALBA8*, *BnALBA11*, *BnALBA13*, *BnALBA20*, *BnALBA22*, *BnALBA23*, *BnALBA24*, and *BnALBA26*, exhibiting moderate to relatively high expression levels (**Figure 6**). The third category consisted of twelve genes, *BnALBA1*, *BnALBA2*, *BnALBA3*, *BnALBA6*, *BnALBA7*, *BnALBA12*, *BnALBA14*, *BnALBA16*, *BnALBA17*, *BnALBA18*, *BnALBA21*, and *BnALBA25*, which showed either minimal expression or tissue-specific patterns (**Figure 6**). Within this third category, five genes, *BnALBA1*, *BnALBA2*, *BnALBA14*, *BnALBA17*, and *BnALBA21*, exhibited specific expression in anthers during the initial flowering and full-bloom stages, with relatively high expression also observed in stamens during these developmental periods (**Figure 6**). These genes additionally displayed moderate expression in inflorescence tips at the initial flowering stage and in buds during bolting. *BnALBA6*, *BnALBA12*, and *BnALBA25* exhibited similar moderate expression patterns in buds at the bolting stage (**Figure 6**). These findings reveal considerable diversity in expression profiles among *BnALBA* family members across different tissues and developmental stages, suggesting potential functional specialization of these genes in various physiological processes of rapeseed.

### Effect of salt and drought stress on the expression of *BnALBAs*

To investigate the potential role of the *BnALBA* gene family in abiotic stress response, qRT-PCR analysis was performed to examine the expression patterns of ten selected genes, comprising five from the RPP20 group and five from the RPP25 group, under salinity and drought stress conditions. These genes were selected from the high- and medium-expression groups based on RNA-seq transcriptome data, as these genes exhibited stable and reliable expression across multiple tissues and developmental stages, allowing a representative assessment of the two subfamilies’ responses to salt and drought stress. The results revealed diverse molecular response patterns among the studied genes, with some showing immediate activation and sustained expression while others exhibited time-specific induction or suppression.

**Figure 7:**
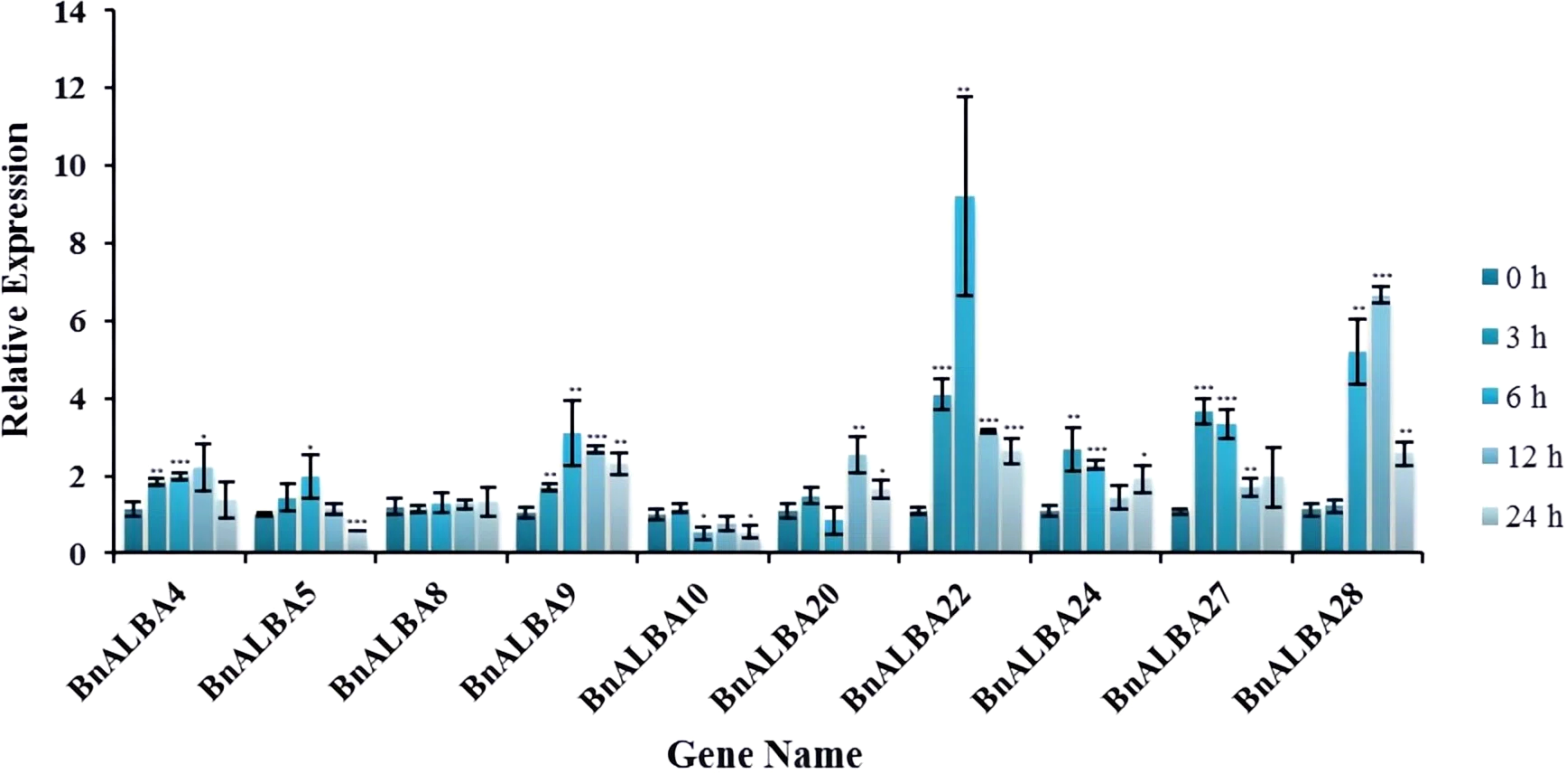
Expression patterns of *BnALBA* genes in response to salt stress. The X- and Y-axes represent relative expression and sampling times, respectively. BnActin7 served as the reference gene for normalization. Asterisks indicate statistically significant differences determined by Student’s t-test (* p < 0.05; ** p < 0.01; *** p < 0.001). Error bars represent standard deviation.

Under salinity stress, *BnALBA8* showed no significant expression changes, while *BnALBA10* was significantly suppressed at 6 and 24 hours post-treatment (**Figure 7**). *BnALBA5* demonstrated suppression at 24 hours. The remaining genes showed significant upregulation at various time points, with *BnALBA22* exhibiting the highest induction (9.22-fold) at 6 hours and *BnALBA28* showing 6.66-fold upregulation at 12 hours (**Figure 7**). *BnALBA22* and *BnALBA9* were identified as the most consistent responders, showing stable and significant upregulation across all time points (**Figure 7**).

**Figure 8:**
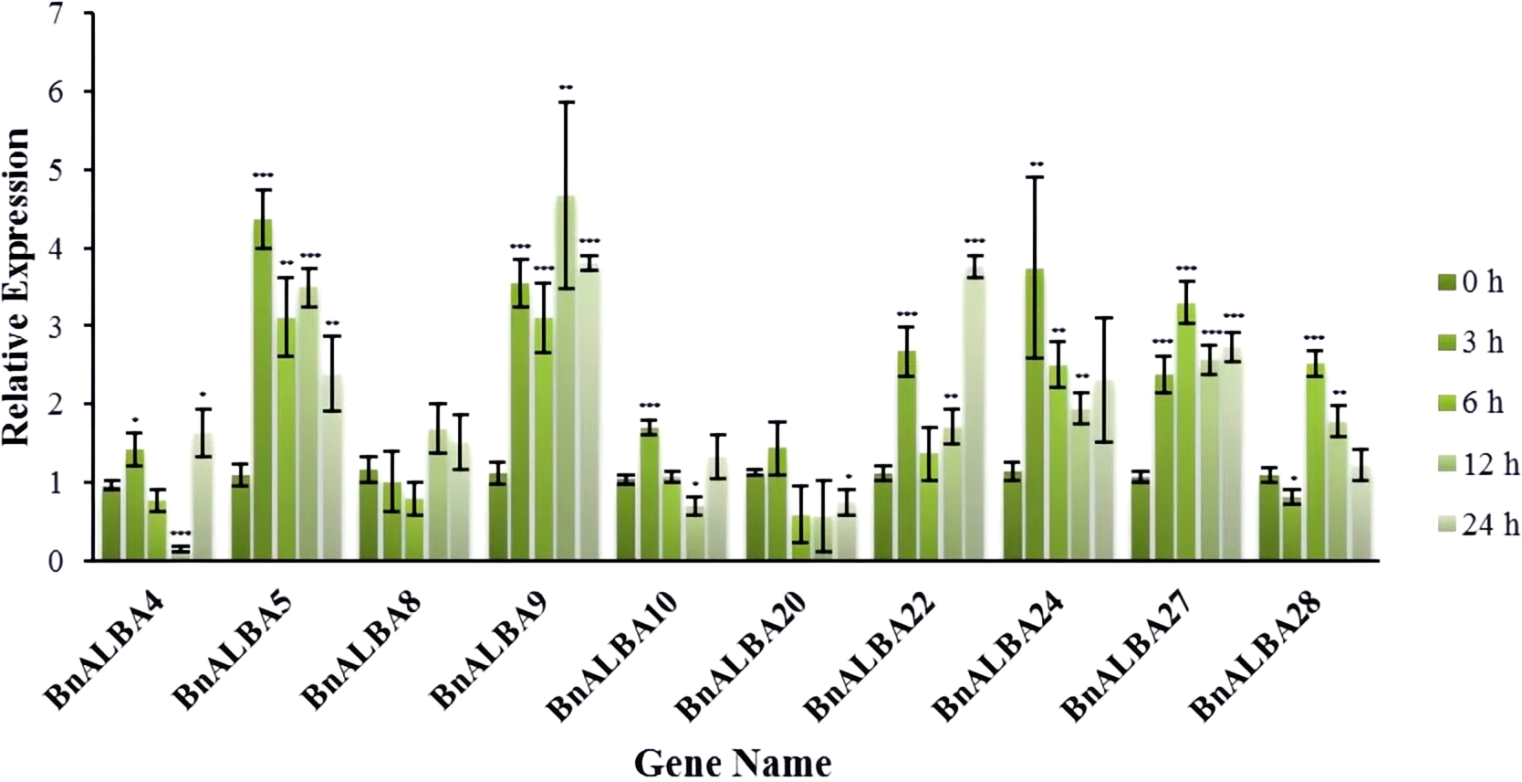
Expression patterns of *BnALBA* genes in response to drought stress. The X- and Y-axes represent relative expression and sampling times, respectively. BnActin7 was used as the reference gene for normalization. Asterisks indicate statistically significant differences based on the Student’s t-test (* p < 0.05; ** p < 0.01; *** p < 0.001). Error bars represent standard deviation.

Under drought stress conditions, *BnALBA8* again showed no significant expression changes, while *BnALBA28* was suppressed at 3 hours (**Figure 8**). *BnALBA10* and *BnALBA4* were suppressed at 12 hours, and *BnALBA20* was suppressed at 24 hours. The other genes showed significant upregulation, with *BnALBA9* reaching 4.66-fold induction at 12 hours and *BnALBA5* showing 4.33-fold upregulation at 3 hours (**Figure 8**). *BnALBA5*, *BnALBA9*, and *BnALBA27* demonstrated consistent and significant upregulation across all time points, indicating a robust response to drought stress (**Figure 8**). Comparative analysis revealed more complex regulation under drought stress compared to salinity stress. While only *BnALBA5* showed both up- and down-regulation under salinity stress, several genes, including *BnALBA4*, *BnALBA10*, *BnALBA20*, and *BnALBA28*, exhibited this bidirectional pattern under drought conditions. It appears that a higher number of *cis*-regulatory elements, including ARE, MYB, and MYC, may play an important role in regulating transcriptional activation under drought and salt stress. For example, *BnALBA22* contains 12 such elements, *BnALBA5*, *BnALBA9*, and *BnALBA27* each harbor 10, while *BnALBA8* has only 6. Genes with more *cis*-elements exhibited stronger induction in response to stress treatments, whereas *BnALBA8* showed no significant change in expression. These observations indicate that the combined presence of ARE, MYB, and MYC elements may increase promoter responsiveness to stress-related signals, which could help to explain the higher transcriptional activation observed for specific *BnALBA* genes under drought and salinity conditions.

## Discussion

Interactions between proteins and nucleic acids play a fundamental role in essential cellular processes. These include replication, transcription, and repair in the case of DNA, and transport, translation, splicing, and silencing in the case of RNA (Cozzolino et al., 2021). Traditionally, nucleic acid-binding proteins were classified into two distinct categories based on their binding specificity for either DNA or RNA (Bartas et al., 2021). However, subsequent research revealed that certain proteins possess the ability to bind both DNA and RNA. A notable example of such dual-specificity proteins is the *ALBA* family.

Research has established that ALBA proteins exist as multi-gene families in plants. For instance, studies have identified 20 *ALBA* genes in maize, 10 in sorghum, 9 in rice, 6 in Arabidopsis, and 7 in grapevine (Verma et al., 2018). Furthermore, 33, 17, and 20 genes encoding ALBA proteins were reported in *Gossypium hirsutum*, *G*. *arboreum*, and *G*. *raimondii*, respectively (Magwanga et al., 2019). The widespread presence of *ALBA* genes across diverse organisms, combined with their multi-genic nature, suggests they play vital roles in various biological processes (Wai et al., 2021). Given the importance of *ALBA* genes, this study aimed to characterize the *ALBA* gene family in rapeseed. Our analysis identified 29 genes encoding ALBA proteins within the rapeseed genome. The BnALBA proteins exhibited considerable variation in their physicochemical properties. Based on the calculated pI values, twelve proteins were classified as acidic and seventeen as basic. An instability index below 40 indicates protein stability under *in vitro* conditions; accordingly, thirteen BnALBA proteins were identified as unstable, while the remaining members of this family were considered stable. Hydropathicity analysis revealed that, except for BnALBA25, all ALBA proteins in rapeseed showed negative GRAVY values, indicating their hydrophilic nature. Furthermore, the aliphatic index suggested that these proteins differ in terms of thermal stability (Lu et al., 2025; Shi et al., 2025a; Yang et al., 2025).

To elucidate the evolutionary history of the *ALBA* gene family in rapeseed, a phylogenetic tree was constructed using protein sequences from both monocot and dicot species, as such trees reflect evolutionary relationships among species and gene families. The results show that the *ALBA* genes in these plants are divided into RPP-20 and RPP-25 subfamilies. Furthermore, within each subfamily, *ALBA* genes from monocot plants of the Gramineae family, including wheat, sorghum, and rice, show close evolutionary relationships, indicating their high sequence conservation within this plant lineage. A similar clustering pattern is observed among the dicot species, including rapeseed, Arabidopsis, potato, and soybean. Within dicot species, *ALBA* genes in rapeseed are most closely related to their counterparts in Arabidopsis, consistent with their common taxonomic affiliation in the Brassicaceae family. These findings align with previous phylogenetic studies on *ALBA* genes across diverse organisms, which classified them into three groups: an archaeal-specific group, and two eukaryotic groups corresponding to RPP-20 and RPP-25. Moreover, evolutionary analyses of the *ALBA* gene family in tomato and rice also support their classification into the RPP-20 and RPP-25 subfamilies, with genes from closely related species grouping together within each subfamily (Goyal et al., 2016; Verma et al., 2018; Wai et al., 2021).

To gain deeper insights into the evolutionary relationships of the *ALBA* gene family, the exon–intron structures of its constituent genes and the conserved motifs of their encoded proteins were analyzed. The results showed that the exon–intron architecture of *BnALBA* genes differs between the two subfamilies, RPP-20 and RPP-25, which is consistent with the phylogenetic analysis. Specifically, *BnALBA* genes in the RPP-20 subfamily contained 3 or 4 introns, while those in the RPP-25 subfamily possessed 6 to 12 introns. A previous study on the *ALBA* gene family in tomato reported a similar pattern: *ALBA* genes belonging to the RPP-20 subfamily had 3 or 4 introns, whereas the number of introns in the RPP-25 subfamily ranged from 5 to 8 (Wai et al., 2021). From an evolutionary standpoint, the difference in intron numbers between the RPP-20 and RPP-25 subfamilies may reflect their distinct evolutionary trajectories (Yaghobi and Heidari, 2023). Moreover, since genes with fewer and shorter introns are generally associated with more efficient responses to environmental changes, it can be inferred that *ALBA* genes in the RPP-20 subfamily may respond more rapidly to environmental fluctuations and stresses (Heyn et al., 2015).

An analysis of conserved motifs and their distribution in the two rapeseed *ALBA* subfamilies RPP-20 and RPP-25 revealed that Motif 1, which is associated with the ALBA domain, was present in all ALBA proteins. However, protein lengths, motif compositions, and their arrangements differed significantly between the two subfamilies. This variation in protein length and conserved motif profiles can be attributed to the presence of RGG/RG repeats in the RPP-25 subfamily (Goyal et al., 2016). Specifically, Motifs 5, 7, 8, 10, 11, 12, and 14, identified exclusively in the RPP-25 subgroup, were found to correspond to RGG/RG repeat motifs. Due to their unique molecular characteristics, RGG/RG repeats are involved in a wide range of cellular processes, including DNA repair, chromatin remodeling, transcription, RNA processing, and translation (Thandapani et al., 2013; Wai et al., 2021). Given the critical roles of these processes in plant responses to environmental stresses, it is plausible that proteins harboring RGG/RG domains also contribute to stress-responsive functions in plants (Bhadouriya et al., 2021; Song et al., 2021).

Fine-tuning of gene expression occurs at both pre- and post-transcriptional levels, primarily mediated by CAREs in the promoter region and microRNAs, respectively, and is crucial for gene function (Jha et al., 2025). CAREs within promoters serve as key units for transcription initiation. Through their interactions with transcription factors and RNA polymerases, they play a central role in regulating gene expression in response to hormones, environmental stresses, light, and plant developmental signals (Schmitz et al., 2022; Archuleta et al., 2024). As these non-coding elements control the spatiotemporal expression of genes across various growth stages, they are critically important in defining gene function in diverse biological processes (Biłas et al., 2016). Accordingly, the promoters of *BnALBA* genes were analyzed, revealing a wide array of CAREs with functions related to circadian rhythm, stress responses, hormone signaling, and growth and development. A key finding was that 80% of the identified CAREs are associated with responses to biotic and abiotic stresses, as well as to hormones such as abscisic acid, gibberellin, auxin, ethylene, and salicylic acid. The presence of CAREs associated with stress and hormone responses has also been documented in the promoters of *ALBA* genes in other plant species, including tomato, rice, sorghum, and Arabidopsis (Verma et al., 2018; Wai et al., 2021).

At the post-transcriptional level, microRNAs (miRNAs), which are short non-coding RNAs, play a critical role in fine-tuning eukaryotic gene expression by directing target mRNAs for degradation or translational repression (Zhao et al., 2025). In rapeseed, bna-miR172a and bna-miR172d directly target *BnALBA18*, bna-miR162a regulates *BnALBA3*, and bna-miR6029 acts on both *BnALBA14* and *BnALBA21*. Research in rapeseed and other plants underscores the fundamental roles of these miRNAs in growth, development, and adaptation to environmental changes. For instance, an integrated mRNA-miRNA transcriptome profiling study of the rapeseed variety S268 under salt stress revealed that bna-miR172a is a key responsive molecule, showing significantly upregulated expression. Furthermore, the target genes of this miRNA are functionally linked to the ABA signaling pathway and the SNARE complex-mediated membrane trafficking pathway, highlighting its mechanistic role in stress adaptation (Liu et al., 2025). This molecule plays a role in tomato resistance to *Phytophthora infestans* (Luan et al., 2018) and in enhancing soybean tolerance to salt stress (Pan et al., 2016). The function of miRNA162a has been demonstrated in rice resistance to the fungus *Magnaporthe oryzae* and in increasing yield (Li et al., 2020). Furthermore, bna-miR6029 is involved in fatty acid biosynthesis during rapeseed seed development and is recognized as a negative regulator of genes related to the nitrogen metabolism pathway in this plant (Wang et al., 2016; Chen et al., 2019). Accordingly, it can be concluded that the precise regulation of *BnALBA* genes expression through the coordinated interaction of CAREs in the promoter and miRNA molecules plays a vital role in rapeseed’s response and adaptation to stresses, as well as in its growth and developmental processes. These results highlight the importance of multi-level gene expression regulation in optimizing plant biological responses.

Gene duplication is a fundamental mechanism for generating novel gene functions and serves as the primary driver for the expansion and diversification of gene families, as well as for evolutionary innovation in plant genomes (Panchy et al., 2016). Although duplicated genes often revert to singletons through the loss of one copy, several alternative evolutionary paths can lead to their retention. These include hypofunctionalization, where both retained copies exhibit reduced expression to collectively fulfill the original function; subfunctionalization, where the copies partition the ancestral gene’s roles; and neofunctionalization, where one copy acquires an entirely new function (Birchler and Yang, 2022). Our results indicate that segmental and whole-genome duplication (WGD) events have been the principal forces behind the expansion of the *BnALBA* gene family, consistent with the history of large-scale genomic duplications in rapeseed (Xu et al., 2025).

These findings are consistent with the evolutionary history of rapeseed, an allopolyploid species derived from the hybridization of *B. rapa* and *B. oleracea*. Its polyploid genome has provided a substrate for large-scale genomic rearrangements, including segmental duplications (Chalhoub et al., 2014; Shi et al., 2025b). Furthermore, the paralogous *BnALBA* genes have evolved under strong purifying selection (mean Ka/Ks value: 0.189), which has led to the conservation of their core functions in gene regulation, RNA metabolism, mRNA translatability, and processes related to growth and stress adaptation. Additionally, genome synteny analysis demonstrated significant collinearity between the *BnALBA* genes and their homologs in *B. rapa*, *B. oleracea*, and Arabidopsis. This high degree of synteny underscores the evolutionary conservation of the *ALBA* gene family and highlights the close phylogenetic relationship among these species within the Brassicaceae family.

Given the intrinsic link between gene expression patterns and gene function, we investigated the spatiotemporal expression profiles of *BnALBA* genes across various tissues and organs at different developmental stages of rapeseed. Furthermore, the transcriptional response of selected *BnALBA* genes to drought and salt stress was analyzed using quantitative reverse transcription PCR (qRT-PCR). Analysis of the expression profiles of all 29 *BnALBA* genes revealed highly diverse and tissue-specific patterns. Categorizing these genes into three groups based on their expression levels, ranging from ubiquitous to highly tissue-specific, suggests that distinct functional specializations exist within the family. Genes with high, broad expression are likely involved in fundamental cellular processes, whereas those with restricted, tissue-specific expression probably fine-tune specific physiological processes, such as flower development and seed formation at particular stages. These findings align with previous reports on *ALBA* genes in other species, including Arabidopsis, tomato, cotton, and rice (Verma et al., 2018; Magwanga et al., 2019; Náprstková et al., 2021; Wai et al., 2021).

For instance, in tomato, several *SlALBA* genes, such as *SlAlba3*, *SlAlba4*, SlAl*b*a6, *SlAlba7*, and *SlAlba8*, were predominantly expressed in vegetative organs like roots and leaves. In contrast, other members, including *SlAlba1*, *SlAlba2*, and *SlAlba5*, exhibited higher expression in reproductive organs such as flowers and fruits. This distinct expression pattern suggests specific roles for *SlALBA* genes in regulating the development and function of different tomato organs, reflecting functional divergence across developmental stages. A similar expression diversity is observed in rice. While *OsAlba1* and *OsAlba7* showed moderate, constitutive expression across all tissues, *OsAlba2*, *OsAlba3*, *OsAlba5*, and *OsAlba9* displayed low or undetectable expression levels. Conversely, *OsAlba4*, *OsAlba6*, and *OsAlba8* were highly expressed in all examined tissues, including roots, stems, leaves, flag leaves, leaf sheaths, and panicles. Notably, *OsAlba4* expression was strongest in roots, whereas *OsAlba8* was more abundant in stems; all three genes also showed elevated expression in flag leaves and panicles (Verma et al., 2018).

Furthermore, studies in Arabidopsis have established that *ALBA* genes play critical roles in male reproductive development and the response to heat stress. Specifically, *ALBA4* and *ALBA6* are vital for RNA metabolism, storage, and/or translational control in pollen under heat stress (Náprstková et al., 2021), while *ALBA3* is essential for protecting male fertility against such stress. In line with these findings, the specific expression of *BnALBA1*, *BnALBA14*, *BnALBA21*, *BnALBA2*, and *BnALBA17* in the anther and stamen during the initial flowering and full-bloom stages, along with the moderate expression of *BnALBA6*, *BnALBA12*, and *BnALBA25* during the bolting stage, suggests a conserved and crucial role of *BnALBA* genes in rapeseed reproductive development.

Plants activate complex molecular networks to respond to environmental stresses, encompassing processes from stress perception and signal transduction to the expression of specific stress-related genes and metabolites (Huang et al., 2012). The presence of ALBA genes across diverse lineages of life, together with their established roles in fundamental processes such as genome packaging, RNA metabolism, and transcriptional and translational regulation, strongly suggests their involvement in plant stress responses (Goyal et al., 2016; Jagadeesh and Vembar, 2024; Ci et al., 2025). This inference is supported by previous research in tomato, rice, and Arabidopsis, where the expression of *ALBA* genes has been shown to be significantly modulated by various abiotic stresses (Verma et al., 2018; Náprstková et al., 2021; Wai et al., 2021).

In the present study, *BnALBA* genes also exhibited differential expression under salt and drought stress conditions. Notably, *BnALBA8* expression remained largely unchanged under these stresses. In contrast, *BnALBA9* and *BnALBA22* were significantly upregulated under salt stress, while *BnALBA5*, *BnALBA9*, and *BnALBA27* showed significant upregulation at all-time points under drought stress. The expression patterns of other genes were more complex: *BnALBA5* under salt stress, and *BnALBA4*, *BnALBA10*, *BnALBA20*, and *BnALBA28* under drought stress, were significantly induced at certain time points and suppressed at others. This pattern of stress-responsive regulation is consistent with findings in other species. For instance, in tomato, *SlAlba4* and *SlAlba5* are induced by heat stress, *SlAlba6* is upregulated in response to salinity, *SlAlba3* responds to drought, and *SlAlba8* is activated under cold stress. Furthermore, with the exception of *SlAlba3*, the expression of these genes was also induced by ABA treatment (Wai et al., 2021).

Significant alterations in *ALBA* gene expression in response to abiotic stresses have also been documented in rice. Specifically, the transcription level of *OsAlba7* was markedly increased under severe salinity, cold, heat, and drought stresses. Furthermore, the expression of *OsAlba3* and *OsAlba9* was upregulated under drought and salinity conditions, supporting their involvement in stress adaptation (Verma et al., 2018). Functional analyses in rice and cotton have provided direct evidence for the role of *ALBA* genes in stress tolerance. In cotton, the genes *GhALBA4* and *GhALBA5* exhibited a strong transcriptional response to salinity and drought. When these genes were silenced using VIGS, the resulting knockout plants displayed heightened sensitivity to salt and drought stress. Compared to wild-type plants, the silenced lines showed reduced antioxidant enzyme activity and elevated levels of oxidative stress, demonstrating the critical function of these genes in the plant’s oxidative stress response (Magwanga et al., 2019).

The rice *OsAlba1* has been identified as a dehydration-responsive gene that likely functions via an ABA-dependent pathway. Functional complementation assays in a ΔPop6 mutant further demonstrated that *OsAlba1* contributes to oxidative stress resistance (Verma et al., 2014). In Arabidopsis, the *ALBA4*, *ALBA5*, and *ALBA6* genes enhance thermotolerance by binding to heat shock factors (HSFs) during heat stress. This interaction stabilizes the HSFs within stress granules (SGs) and P-bodies (PBs), thereby preventing their degradation (Tong et al., 2022). SGs and PBs play key roles in post-transcriptional regulation under stress conditions. SGs protect mRNAs by storing untranslated transcripts, while PBs are involved in mRNA decay and turnover, together ensuring proper mRNA stability and translation control during stress responses (Adjibade and Mazroui, 2023; Huang et al., 2024). Studies indicate that RNA-binding proteins, such as ALBA proteins, play important roles in the formation of SGs and PBs (Jagadeesh and Vembar, 2024). Therefore, it can be suggested that genes such as *BnALBA5*, *BnALBA9*, *BnALBA22*, and *ALBA27* may contribute to mRNA stability and translation regulation under stress conditions such as salinity and drought. By modulating their expression in response to various stresses, these genes may play important roles in enhancing plant tolerance. Further experimental functional characterization is thus essential to validate their predicted roles and to elucidate the molecular mechanisms underlying plant resilience to environmental challenges.

## Conclusion

This study presents the first comprehensive analysis of the *ALBA* gene family in rapeseed, identifying 29 genes unevenly distributed across chromosomes. Evolutionary analyses revealed that segmental and whole-genome duplications (WGD) were the main forces driving the expansion of this family while maintaining their conserved biological functions. These genes were classified into two subfamilies, RPP-20 and RPP-25, which display distinct exon–intron structures and motif compositions, suggesting potential functional divergence. CAREs associated with stress, hormone signaling, growth and development, and circadian regulation, together with post-transcriptional regulation by miRNAs, highlight their central roles in rapeseed growth and stress adaptation. Transcriptomic analyses across developmental stages further showed that *ALBA* genes exhibit diverse and tissue-specific expression patterns, reflecting their precise and versatile regulatory functions. In addition, qRT-PCR analysis demonstrated that several *BnALBA* genes respond significantly to abiotic stresses, with *BnALBA9* and *BnALBA22* strongly induced by salt stress, and *BnALBA5*, *BnALBA9*, and *BnALBA27* consistently upregulated under drought stress. While this study provides a comprehensive identification and expression profiling of the *ALBA* gene family in rapeseed, further research is needed to deepen our understanding of their precise biological functions. Future investigations integrating functional genomics, proteomics, and metabolomics, along with genetic engineering approaches such as CRISPR/Cas9-mediated gene editing or overexpression, will help validate the roles of *BnALBA* genes in plant development and stress adaptation.

## Data Availability

The original contributions presented in the study are included in the article/**Supplementary Material**. Further inquiries can be directed to the corresponding author.

## Supplementary material

Supplementary File 1 Primers used in the qRT-PCR analysis of selected *BnALBA* genes.

Supplementary File 2 General information on the *ALBA* gene family identified in the rapeseed genome.

Supplementary File 3 Conserved motifs in the *ALBA* gene family of rapeseed.

Supplementary File 4 Ka, Ks, and Ka/Ks parameters and selective pressure on duplicated *BnALBA* gene pairs.
