# Supplementary material for: Genome-wide identification and characterization of the ALBA gene family in rapeseed (Brassica napus L.) and its role in development and abiotic stress responses
Source: Front Plant Sci. 2026 Jan 5;16:1721794. doi: 10.3389/fpls.2025.1721794 (PMC12812609; doi:10.3389/fpls.2025.1721794)
Supplement: Supplementary File 1 — Primers used in the qRT-PCR analysis of selected BnALBA genes. [file Table1.docx]

| **Gene Name** | **Forward** | **Revers** |
| --- | --- | --- |
| *BnALBA4* | GTCGAATTGTCTGCCCTTGG | TCTCTGACTTGCCAAGCGTA |
| *BnALBA5* | AGAGGCAGAGCAAGAAGAGG | TGTGGAGGAGGAGCATTGTT |
| *BnALBA8* | GGGATCACGGAAGGAGTCAA | GCCATTCCAAGTGCAGACAA |
| *BnALBA9* | AAGAGGAAGAGGGAGGGGAA | TGTACCCTCCTCTTCCTCGA |
| *BnALBA10* | TTGTCTGCACTTGGAATGGC | CAGACTTGGCGAGCGTTATC |
| *BnALBA20* | TACATGCAGCAGTACACCGA | TCCTCTTGAATCGCCCTTGA |
| *BnALBA22* | AAGATGGAGGTTGGGAACGT | CCACGGTGTTCATGAGTAGC |
| *BnALBA24* | CCCAACAAGACGGAGGAAAC | CACCGTATCCGTAGTCTCCC |
| *BnALBA27* | CTCCTCCTCATGAACACGGT | GACCCTCATAACCACCACCA |
| *BnALBA28* | TTCTACGTCAACCTCGCCAA | TAGGTTTCTGCACAGGACGT |
| *BnActin7* | ACAGTGTCTGGATCGGTGGTTC | TGCCTCATCATACTCAGCCTTG |

Supplementary File 1. Primers used in the qRT-PCR analysis of selected BnALBA genes.
